# Pharmacokinetics of tenvermectin in swine, a novel antiparasitic drug candidate—comparison with ivermectin

**DOI:** 10.1002/vms3.1085

**Published:** 2023-02-11

**Authors:** Guiyu Li, Xingyuan Cao, Jianwei Liao, Yanming Wei

**Affiliations:** ^1^ Department of Veterinary Pharmacology and Toxicology Gansu Agricultural University Lanzhou Gansu China; ^2^ Department of Veterinary Pharmacology and Toxicology College of Veterinary Medicine China Agricultural University Beijing China; ^3^ Department of Research and Development Shisenhai (Hangzhou) Pharmaceutical Technology Co., Ltd. Hangzhou Zhejiang China

**Keywords:** antiparasitic drug, ivermectin, pharmacokinetics, swine, tenvermectin

## Abstract

Tenvermectin (TVM) is a novel 16‐membered macrolide compound isolated and purified from the fermentation broth of genetically engineered Streptomyces avermitilis strain *MHJ1011*. TVM and ivermectin were administered at the dose of 0.3 mg/kg body weight through a single subcutaneous injection route followed by plasma collectiom and analysis at different time intervals. Plasma concentrations of TVM and IVM were determined by high‐performance liquid chromatography with fluorescence detector. Pharmacokinetic analysis was completed using the non‐compartmental method with WinNonlin™ 6.4 software. TVM is rapidly absorbed after administration with peak plasma concentrations (*C*_max_, 9.78 ± 2.34 ng/ml) obtained within 6–22 h. AUC_0‐last_ was 586.86 h·ng/ml ± 121.24 h·ng/ml. The mean elimination half‐life of TVM (*T*_1/2λz_) was 97.99 h ± 46.41 h. The *T*_1/2λz_ of IVM was 146.59 h ± 22.26 h in the study. The present study showed that subcutaneous administration of TVM at 0.3 mg/kg body weight (BW) in swine is absorbed more rapidly than IVM in swine. Compared to the pharmacokinetic characteristics of IVM, there was little difference in the half‐life of TVM among different individuals. The data will contribute to refining the formulation and dosage regime for TVM administration.

## Graphical abstract

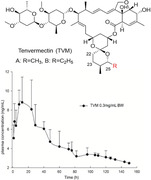

## 1 INTRODUCTION

**FIGURE 1:**
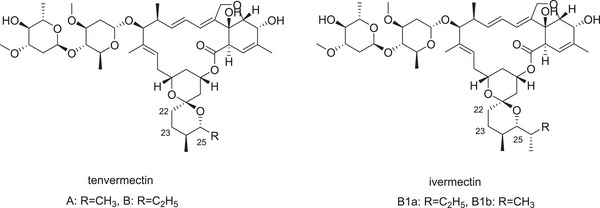
Structures of tenvermectin and ivermectin

Veterinary antiparasitic drugs, represented by ivermectin (IVM), have been widely used in clinical practice (Ge et al., 2022; Wan et al., 2017; Wang et al., 2016; Zhang et al., 2019; Zhang et al., 2022), but IVM has a long metabolic cycle leading to a large amount of drug residues in the faeces (Mckellar & Gokbulut, 2012); it slows down the rate of treating the animal manure by insects such as dung beetle (Ambrožová et al., 2021). Tenvermectin (TVM) is a novel 16‐membered macrolide compound isolated and purified from the fermentation broth of genetically engineered *Streptomyces avermitilis strain MHJ1011* (Wan et al., 2017; Zhang et al., 2019). TVM is composed of two components: TVM A (25‐methyl‐22,23‐dihydro avermectin; R = CH_3_) and TVM B (25‐ethyl‐22,23‐dihydro avermectin; R = C_2_H_5_) (Ge et al., 2022) (Figure 1). There is a difference at the C25 site between these structures, with a methyl group for TVM A and an ethyl group for TVM B. The composition of TVM A and TVM B in fermentation products is nearly 3:1.

Avermectin, IVM and milbemycin are well‐known 16‐membered macrolide compounds (Gaisser et al., 2003). IVM, a semisynthetic derivative of the avermectin family, has two components, B1a (>80%) and B1b (Lifschitz et al., 1999); they have more alkane side chains than TVM A and B in C25. Recently, TVM has been used as a potential pesticide and an antiparasitic drug (Fei et al., 2018; Zajíčková et al., 2020). It has been reported that TVM exhibits better pharmacological properties against *Tetranychus cinnabarinus* and *Bursaphelenchus xylophilus* than IVM or milbemycin (Arieta‐Román et al., 2010; Deng et al., 2019; Marty et al., 2005; Zhou et al., 2012). In addition, TVM showed an enhanced activity against *trichuriasis* compared with IVM (Huang et al., 2015).

Although the primary structure of TVM is very similar to that of IVM, it is necessary to conduct a series of studies as it is a completely new structure. We report for the first time a plasma pharmacokinetic study of TVM in pigs following a single subcutaneous administration at a dose of 0.3 mg/kg body weight (BW). This dose was the same concentration as the commercial formulation Ivomec^®^.

## 2 MATERIALS AND METHODS

### 2.1 Animal

This study was approved by the Institutional Committee on Animal Care and Use at Chinese Agricultural University (Approval number *12205‐15‐D‐007*). The pharmacokinetic study of TVM was determined in 12 clinically healthy Danish Landrance × Yorkshire × Duroc swine (weight 27.5 kg ± 2.5 kg). They were divided into two groups and housed in separate metal cages. Twelve pigs, half male and half female, were used in this study. All swine were kept in a constant temperature (25°C) and humidity (60%) environment, and each pig was given sufficient feed and light in as approved by the Institutional Animal Care and Use Committee of Chinese Agricultural University (Beijing, China). The study was performed after 5 days entering the experimental animal room. All pigs are exposed to a 12 h artificial light‐dark cycle and kept in cages with free access to water and standard laboratory feed. Every pig was evaluated physically before the start of the experiment, and venous blood was collected as a blank sample. All swine were healthy during the rearing process. Before the experiment they were divided into two parts, six pigs (three males and three females) were used for the phmarmacokinetic test of TVM and the other six pigs (three males and three females) were used for the pharmacokinetic test of IVM.

### 2.2 Blood sample collection

Injectable formulations of TVM (10 mg/ml in a mixed solution of propyl gallate 0.01%, disodium edetate 0.02%, 85% water, glycerol 9%, and propylene glycol 5.97%) were provided by Zhejiang Hisun Pharmaceutical Co., Ltd. Prior to the study, the protocols were reviewed and approved by the Institutional Animal Care and Use Committee of Chinese Agricultural University (Beijing, China). IVM injection was used Ivomec^®^ (IVM, No. BE136/13, Merial Animal Health Co., Ltd). Blood samples were collected from the jugular vein into heparinised tubes at 0, 1, 2, 4, 8, 12, 24, 36, 40, 48, 60, 68, 72, 76, 82, 96, 106, 110, 120, 126, 132, 144, 150 and 156 h after subcutaneous administration of 0.3 mg/kg BW of TVM. The samples were centrifuged at 2280 × *g* for 10 min. The obtained plasma samples were each stored at −20°C until analysed.

IVM injection was administered as a single dose of 0.3 mg/kg BW via subcutaneous injection in the neck. Blood samples were taken at 0, 1, 2, 4, 8, 12, 24, 29, 40, 48, 60, 72, 76, 82, 96, 106, 110, 120, 126, 132, 144, 150, 156, 168, 192, 216, 240, 264, 288, 312, 336, 384, 432 and 480 h. The samples were centrifuged at 2280 × *g* for 10 min. The obtained plasma samples were each stored at −20°C until analysed.

### 2.3 Plasma sample analysis

With reference to the drug metabolism assay for IVM, we have developed an assay for TVM (Montigny et al., 1990). Plasma concentrations of TVM were determined using high‐performance liquid chromatography (HPLC, Waters 2695; Waters Crop, USA) and fluorescence detector (Waters 474 fluorescence detector, Waters Associates, Milford, MA, USA). Briefly, 1 ml of acetonitrile (LC grade; Fair Lawn, NJ, USA) was added to 1 ml of plasma. After vortexing for 10 min, the samples were centrifuged at 13,400 *× g* for 5 min. The supernatant of each sample was purified using an Oasis HLB cartridge (3 cc, 60 mg) preconditioned with methanol (3 ml) and water (3 ml). After the supernatant was loaded, 2 ml of water was used to remove the impurities. One millilitre of acetonitrile was applied to obtain the eluate, which was evaporated to dryness under a gentle nitrogen stream at 50°C. The residue was derivatised by dissolving in 100 μl of N‐methylimidazole solution (Aladdin Industrial Corporation, Shanghai, China) in acetonitrile (1:1, *v/v*) and vortexed for 10 s. One hundred fifty microlitres of trifluoroacetic anhydride solution (Aladdin Industrial Corporation, Shanghai, China) in acetonitrile (1:2, v/v) was added and mixed for ≥10 s. After derivatisation in the dark for 30 min, 750 μl of methanol was added to terminate the reaction before automatic injection into the HPLC system. The separation was achieved using a reverse C18 column (4.6 mm × 250 mm i.d., 5 μm particle size), in a column oven at 25°C. Acetonitrile was used as the mobile phase at a flow rate of 1 ml/min. The fluorescent derivative of TVM was detected at an excitation and emission wavelength of 365 and 475 nm, respectively. Each sample was sampled twice and the average was collected. Chromatographic separation conditions for IVM and post‐treatment of blood samples were carried out using the same method as TVM.

### 2.4 Pharmacokinetic analysis

Non‐compartmental analysis analysis was performed by using Phoenix WinNonlin software version 8.1. The following parameters were determined: Terminal rate constant or terminal slope of the concentration‐versus‐time curve (*λz* (1/h)), half‐life or apparent elimination half‐life (*T*_1/2λz_ (h)), time to maximum concentration (*T*_max_), maximum plasma concentration (*C*_max_), area under the concentration‐versus‐time curve from time 0 h to the last measured concentration (AUC_0‐last_), area under the concentration‐versus‐time curve from time 0 h to infinity (AUC_0‐∞_), volume of distribution corrected for bioavailability or volume of distribution per fraction absorbed (Vd/F), clearance corrected for bioavailability (Cl/F), mean residence time (MRT_last_ (h)). The values of all PK parameters were calculated as mean ± standard deviations, and the Mann‐Whitney non‐parametric tests were applied to verify differences between groups by using SPSS Statistics software. *p* Value of less than 0.05 was considered to be statistically different.

### 2.5 Method validation

The calibration curve was plotted by assaying the calibration samples at five concentration levels (2.5, 5, 10, 50, 100 ng/ml). Typical equations for the calibration curves and the correlation coefficients (*r*) were *y* = 13,913*x*‐17,595 (*R* ^2^ = 0.9971) for TVM, and *y* = 24,481*x*‐10,459 (*R* ^2^ = 0.9987) for IVM. The limit of detection (LOD) and limit of quantification (LOQ) were defined as 3 × S/N (signal/noise) and 10 × S/N. Recovery was used to express accuracy and precision was determined as the relative standard deviation (RSD) of the mean recovery for reproducibility (intra‐day, *n = 3*) and intermediate precision (inter‐day, three consecutive days, *n = 3*) analyses.

## 3 RESULTS

All swine received a single dose of TVM or IVM (0.3 mg/kg BW) administered via the subcutaneous route of administration. All the animals remained healthy throughout the study. No adverse reactions were observed following the subcutaneous administration of TVM.

In the concentration range of 1–100 ng/ml, the calibration curves for TVM or IVM in plasma were linear. The correlation coefficients (Figures S1 and S2) demonstrated good linearity over a wide concentration range. The lower limits of quantification of the assay, expressed as LOD and LOQ, were determined by determining the lowest concentration in the standard curve that could be quantified with 80%–120% accuracy and precision (variation coefficient 20%). The LOD and LOQ of TVM were 0.5 and 1 ng/ml, respectively. The inter‐day and intra‐day coefficients of variation at three different concentrations (1, 10 and 50 ng/ml) were all <10.74%, while the mean recoveries ranged from 86.15% to 111.57% (Tables S1 and S2). IVM and TVM have the same LOD and LOQ. The recoveries of the three spiked concentrations of IVM ranged from 82.88% to 104.32%. The intra‐day coefficients of variation were less than 9.04% and the inter‐day coefficients of variation were less than 5.35% (Tables S3 and S4).

**FIGURE 2:**
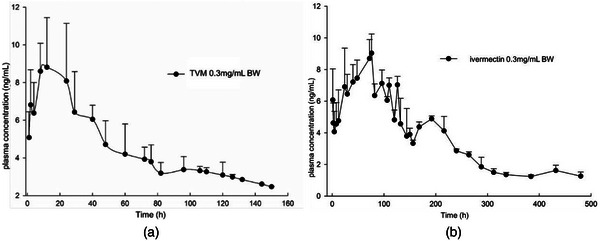
Plasma concentration‐time curve of tenvermectin (a) and ivermectin (b) after i.h.

The plasma concentration‐versus‐time curves of TVM in swine were plotted on a semilogarithmic plot (Figure 2, Table S5). TVM in plasma reached a peak at approximately 14.00 h ± 7.90 h after subcutaneous administration in swine. The peak value was lower than that of IVM (*T*_max_, 57.33 h ± 25.88 h) in swine. The pharmacokinetic parameters (mean ± SD) are presented in Table 1. In this study, IVM was also administered 0.3 mg/kg BW. The results of the present study show that subcutaneous administration of TVM at 0.3 mg/kg BW in swine is absorbed more rapidly than IVM in swine (Table S6). The maximum concentration (*C*_max_) and the area under the receiver operating characteristic curve (AUC) from dosing to the time of the last measured concentration ≥ LOQ of that dosing period (AUC_0‐last_) of TVM were approximately half of those of IVM. The mean elimination half‐life of TVM (*T*_1/2λz_) was 97.99 ± 46.41 h and half‐life of IVM was 148.16 ± 57.02 h, suggesting that the drug was cleared quickly from swine with the drug being detectable in plasma.

**TABLE 1:** Pharmacokinetic parameters of tenvermectin and ivermectin in swine after single subcutaneous administration at a dose of 0.3 mg/kg BW (*n* = 6)

Pharmacokinetic parameter	Tenvermectin	Ivermectin	*p* Value
***λz* (1/h)**	0.0085 ± 0.0042	0.0048 ± 0.0007	0.0433
***T*_1/2λz_ (h)**	97.99 ± 46.41	146.59 ± 22.26	0.0411
***T*_max_ (h)**	14.00 ± 7.90	57.33 ± 25.88	0.013
***C*_max_ (ng/ml)**	9.78 ± 2.34	10.14 ± 0.36	>0.9999
**AUC_0‐last_ (h·ng/ml)**	586.86 ± 121.24	1728.98 ± 94.13	0.0022
**AUC_0‐∞_ (h·ng/ml)**	975.53 ± 194.09	1997.29 ± 139.92	0.0022
**Vd/F (ml/kg)**	42578.68 ± 13736.30	31842.55 ± 5111.64	0.2403
**Cl/F (ml/h/kg)**	322.82 ± 72.74	150.84 ± 10.90	0.0022
**MRT_last_ (h)**	47.65 ± 11.74	161.37 ± 1.72	0.0022

*Note*: *λz*, terminal rate constant or terminal slope of the concentration‐versus‐time curve; *T*_1/2λz_, half‐life or apparent elimination half‐life; *T*_max_, time to maximum concentration; *C*_max_, maximum plasma concentration; AUC_0‐last_, area under the concentration‐versus‐time curve from time 0 h to the last measured concentration; AUC_0‐∞_, area under the concentration‐versus‐time curve from time 0 h to infinity; Vd/F, volume of distribution corrected for bioavailability or volume of distribution per fraction absorbed; Cl/F, clearance corrected for bioavailability; MRT_last_, mean residence time. Data were presented as mean ± SD (*n* = 6). *p* Value of less than 0.05 was considered to be statistically different.

## 4 DISCUSSION

To our knowledge, this is the first study to report the pharmacokinetics of TVM in swine following subcutaneous injection. The TVM recoveries, the inter‐day precision and intra‐day precision were found were found in the range of 94%–105%, 1.05%–10.74% and 1.40%–2.99%. Acceptable ranges of accuracy and precision are within 15% of the actual value, respectively.

Compared to the pharmacokinetic profile of IVM, the half‐life of TVM is shorter, only 67% of that of IVM (Mckellar & Gokbulut, 2012). The average *T*_max_ of TVM was 14.00 ± 7.90 h, while IVM reported values of 57.33 ± 25.88 h. At the same time, compared with IVM, the inter‐individual variation of each parameter of TVM is smaller. The blood concentration of TVM can be lower than 6 μg/ml after 48 h of administration, while IVM remains above this concentration at 126 h.

Previous studies found significant differences in IVM kinetics on swine of different species, age and weight (Craven et al., 2002a; Craven et al., 2002b; Lifschitz et al., 1999; Lo et al., 1985). Differences in body composition (fat or lean) and growth period (growth or maintenance) of pigs have been found in previous studies to affect the kinetic parameters of macrolides (Mckellar & Gokbulut, 2012). The high lipophilicity of macrolides, the large amount of fat residues and the important role of fat metabolism suggest that the fat composition of the administered animals may influence the pharmacokinetics of these drugs (Mckellar & Gokbulut, 2012). It has also been observed that plasma kinetic disposition of IVM and moxidectin was markedly affected by body weight of pigs after subcutaneous administration (Mckellar & Gokbulut, 2012). In obese pigs, the *T*_1/2λz_ and MRT_last_ of IVM were significantly longer and the *C*_max_ values were lower, which the authors speculate is due to the higher adipose tissue content and increased tissue distribution in fat pigs compared to lighter pigs (Craven et al., 2002a).

Similar to their previously IVM metabolism parameters (*T*_max,_ 22–75 h) (Craven et al., 2002b; Craven et al., 2010; Lifschitz et al., 1999; Lo et al., 1985; Scott & Mckellar, 1992), our study confirmed that the *T*_max_ of IVM was 57.33 ± 25.88 h (Mckellar & Gokbulut, 2012). The main reason for the discrepancy in the data could be the weight of the pigs. Despite some differences in these parameters (Craven et al., 2002a,b; Craven et al., 2010; Lifschitz et al., 1999; Scott & Mckellar, 1992), this experimental results still show that TVM was metabolised at a faster rate in vivo (*T*_max_ of TVM was 14.00 ± 7.90 h). The advantage of TVM in metabolic rate may help it to have a shorter rest period in use.

The main reasons for the rapid metabolism of TVM in vivo are attributed to structure and the ratio of TVM A to TVM B. Macrolide drugs have structural features that affect their pharmacokinetics. The most significant difference between moxidectin and IVM is that the former has a bisoleandrosyloxy disaccharide on C‐13, while the latter does not. This difference results in the highly lipophilic nature of moxidectin, which gives it a longer half‐life and a slower reduction in AUC (Craven et al., 2010). These results were consistent with a change in the metabolic status of TVM and IVM in vivo. The ratio of TVM A and B was approximately 3:1, while IVM B1a comprises more than 80% of the drug formulation. Compared to TVM, IVM B1a has a longer side chain, which may lead to higher lipid solubility of IVM (Errecalde et al., 2021). The aqueous solubility of an active ingredient as well as the characteristics of its pharmacotechnical preparation can affect systemic availability. The vehicle in which the drug is formulated may play a role in the absorption kinetics and the resultant plasma availability. Another reason for the short half‐life of TVM (9% propylene glycol and 6% glycerol) is that TVM may use fewer non‐aqueous solvents than IVM. Previous reports have shown that the nature of the formulation used to deliver the active macrolide fundamentally alters its pharmacokinetic behaviour. Even minor modifications to macrolide formulations can lead to changes in plasma configuration, their concentration and residence time at the parasite site (Craven et al., 2010; Lanusse et al., 1997; Lifschitz et al., 2000; Wicks et al., 1993).

The value of AUC was smaller than that of IVM (1713.8 h·ng/ml) in swine (Scott & Mckellar, 1992). Further studies should be conducted to verify the safety and effectiveness of TVM in pigs.

Also TVM metabolism is faster resulting in a potentially less toxic profile. Several experimental studies have been performed to assess the clinical efficacy and safety profile of TVM. The acute toxicity of TVM (LD_50_ = 74.41 mg/kg) is lower than that of IVM (LD_50_ = 53.06 mg/kg) as determined by the oral acute toxicity test in mice. Ames test results of TVM were all negative for *Salmonella typhimurium* TA97a, TA98, TA100, TA102 and TA1535 with and without the metabolic activation system, indicating a lack of mutagenic activity (Fei et al., 2018). The reported experimental data indicate the excellent efficacy of both TVM and IVM against *Ascaris suum* with a single subcutaneous injection 0.3 mg/kg BW, with a potential effect on *Trichuris suis* by only TVM. Given the efficacy of TVM against swine nematodes (Fei et al., 2018; Chaccour et al., 2017), it has potential value as an important new antiparasitic drug against swine nematodes.

The objective of this prospective study was to report the pharmacokinetic profile of TVM in swine following its subcutaneous administration. This study found that TVM was more rapidly distributed when applied subcutaneously to the same dose of IVM. However, further studies are needed to determine the appropriate dosing frequency and clinical efficacy of TVM in this species.

## AUTHOR CONTRIBUTIONS

Yanming Wei, Guiyu Li and Xingyuan Cao contributed to study design and execution and gave final approval of the manuscript. Guiyu Li and Xingyuan Cao contributed to data analysis. Guiyu Li and Jianwei Liao donated the test drugs and contributed to technical assistance. Guiyu Li was involved in study execution, data analysis and interpretation and manuscript preparation.

## CONFLICT OF INTEREST

The authors declare that they have no known competing financial interests or personal relationships that could have appeared to influence the work reported in this paper.

### ETHICS STATEMENT

The author confirms that the ethical policies of the journal, as noted on the journal's author guidelines page, have been adhered to and the appropriate ethical review committee approval has been received. The authors confirm that they have adhered to international standards for the protection of animals used for scientific purposes.

### PEER REVIEW

The peer review history for this article is available at https://publons.com/publon/10.1002/vms3.1085.

## Supporting information

Supporting Information

## Data Availability

The data sets generated during and/or analysed during the current study are available from the corresponding author on reasonable request.
