# Supplementary material for: Pharmacokinetics of tenvermectin in swine, a novel antiparasitic drug candidate—comparison with ivermectin
Source: Vet Med Sci. 2023 Feb 11;9(3):1211–6. doi: 10.1002/vms3.1085 (PMC10188071; doi:10.1002/vms3.1085)

**Pharmacokinetics of Tenvermectin in swine, a novel antiparasitic drug candidate—comparison with Ivermectin**

**—Supplementary Information**

**Table captions**

Table S1 - Recovery of different batches of plasma samples spiked with tenvermectin (*n = 3*).

Table S2 - Recovery and coefficient of variation of tenvermectin standard spiked with pig plasma (*n = 3*).

Table S3 - Recovery of different batches of plasma spiked with ivermectin (*n = 3*).

Table S4 - Recovery and coefficient of variation of ivermectin standard spiked with pig plasma (*n = 3*).

Table S5 - Plasma concentration-time curve of tenvermectin after s.c.

Table S6 - Plasma concentration-time curve of ivermectin after s.c.

Table S1 - Recovery of different batches of plasma samples spiked with tenvermectin (*n = 3*).

| Final concentration  (ng/mL) | Batch | 1 day | 2 day | 3 day |
| --- | --- | --- | --- | --- |
| 1 | 1 | 94.73 | 106.78 | 98.76 |
|  | 2 | 100.06 | 100.88 | 97.36 |
|  | 3 | 105.57 | 102.34 | 100.05 |
| 10 | 1 | 90.04 | 105.95 | 98.18 |
|  | 2 | 104.86 | 87.46 | 96.80 |
|  | 3 | 89.71 | 89.55 | 86.15 |
| 50 | 1 | 95.54 | 111.57 | 110.10 |
|  | 2 | 97.18 | 109.26 | 109.40 |
|  | 3 | 100.86 | 110.42 | 101.42 |

Table S2 - Recovery and coefficient of variation of tenvermectin standard spiked with pig plasma (*n = 3*).

| Final concentration  (ng/mL) | Day | Average  recovery | Inter-day  precision | Intra-day  precision |
| --- | --- | --- | --- | --- |
| 1 | 1 | 100.12 | 5.41 | 2.99 |
|  | 2 | 103.33 | 2.98 |  |
|  | 3 | 98.72 | 1.37 |  |
| 10 | 1 | 94.87 | 9.12 | 2.25 |
|  | 2 | 94.32 | 10.74 |  |
|  | 3 | 93.71 | 7.02 |  |
| 50 | 1 | 97.86 | 2.78 | 1.40 |
|  | 2 | 110.42 | 1.05 |  |
|  | 3 | 106.97 | 4.50 |  |

Table S3 - Recovery of different batches of plasma samples spiked with ivermectin (*n = 3*).

| Final concentration  (ng/mL) | Batch | 1 day | 2 day | 3 day |
| --- | --- | --- | --- | --- |
| 1 | 1 | 87.98 | 104.32 | 96.86 |
|  | 2 | 86.56 | 102.13 | 101.17 |
|  | 3 | 86.10 | 103.61 | 103.15 |
| 10 | 1 | 82.88 | 92.02 | 101.70 |
|  | 2 | 96.74 | 83.83 | 100.64 |
|  | 3 | 97.90 | 99.84 | 94.02 |
| 50 | 1 | 90.29 | 97.32 | 97.44 |
|  | 2 | 103.80 | 87.47 | 92.57 |
|  | 3 | 99.58 | 90.78 | 92.73 |

Table S4 - Recovery and coefficient of variation of ivermectin standard spiked with pig plasma (*n = 3*).

| Final concentration  (ng/mL) | Day | Average  recovery | Inter-day  precision | Intra-day  precision |
| --- | --- | --- | --- | --- |
| 1 | 1 | 86.88 | 1.13 | 4.66 |
|  | 2 | 103.35 | 1.08 |  |
|  | 3 | 100.39 | 3.20 |  |
| 10 | 1 | 92.51 | 9.04 | 5.35 |
|  | 2 | 91.90 | 8.71 |  |
|  | 3 | 98.79 | 4.21 |  |
| 50 | 1 | 97.89 | 7.07 | 1.10 |
|  | 2 | 97.86 | 5.46 |  |
|  | 3 | 94.25 | 2.94 |  |

Table S5 - Plasma concentration-time curve of tenvermectin after s.c.

| Time  (h) | Animal number (ng/mL) | | | | | | Mean | SD |
| --- | --- | --- | --- | --- | --- | --- | --- | --- |
|  | 1♂ | 2♂ | 3♂ | 4♀ | 5♀ | 6♀ |  |  |
| 1 h | 4.53 | 5.11 | 7.69 | 4.13 | 5.12 | 3.89 | 5.08 | 1.37 |
| 2 h | 5.27 | 4.76 | 8.33 | 9.64 | 6.71 | 6.18 | 6.82 | 1.86 |
| 4 h | 4.82 | 5.45 | 8.83 | 5.18 | 7.90 | 6.14 | 6.38 | 1.62 |
| 8 h | 9.14 | 7.58 | 11.13 | 7.70 | 8.95 | 7.09 | 8.60 | 1.48 |
| 12 h | 10.93 | 6.19 | 9.65 | 6.42 | 12.56 | 7.09 | 8.81 | 2.64 |
| 24 h | 11.79 | 7.12 | 9.22 | 8.52 | 9.21 | 2.65 | 8.08 | 3.06 |
| 29 h | 7.41 | 6.30 | 5.06 | 8.40 | 8.48 | 2.94 | 6.43 | 2.15 |
| 40 h | 5.88 | 5.17 | 5.36 | 6.91 | 6.95 | 6.05 | 6.05 | 0.75 |
| 48 h | 6.72 | 3.42 | 4.08 | 5.18 | 5.27 | 3.59 | 4.71 | 1.26 |
| 60 h | 3.52 | 3.35 | 3.20 | 7.32 | 4.52 | 3.30 | 4.20 | 1.60 |
| 72 h | 3.50 | 4.20 | 3.36 | 4.91 | 4.32 | 3.31 | 3.93 | 0.64 |
| 76 h | 2.70 | 3.10 | 3.46 | 5.12 | 4.43 | 3.99 | 3.80 | 0.89 |
| 82 h | 2.49 | 2.96 | 3.12 | 3.85 | 3.88 | 2.81 | 3.19 | 0.57 |
| 96 h | 3.32 | 2.64 | 2.60 | 4.37 | 3.55 | 3.79 | 3.38 | 0.69 |
| 106 h | BQL | BQL | BQL | 3.58 | 3.13 | 3.25 | 3.32 | 0.23 |
| 110 h | BQL | BQL | BQL | 3.34 | 3.44 | 3.03 | 3.27 | 0.21 |
| 120 h | BQL | BQL | BQL | 3.82 | 2.98 | 2.47 | 3.09 | 0.68 |
| 126 h | BQL | BQL | BQL | BQL | 3.08 | 2.90 | 2.99 | 0.13 |
| 132 h | BQL | BQL | BQL | BQL | 2.85 | BQL | 2.85 | 0 |
| 144 h | BQL | BQL | BQL | BQL | 2.61 | BQL | 2.61 | 0 |
| 150 h | BQL | BQL | BQL | BQL | 2.48 | 2.46 | 2.47 | 0.02 |
| 156 h | BQL | BQL | BQL | BQL | BQL | BQL | - | - |

BQL: <LOQ

Table S6 - Plasma concentration-time curve of ivermectin after s.c.

| Time  (h) | Animal number (ng/mL) | | | | | | Mean | SD |
| --- | --- | --- | --- | --- | --- | --- | --- | --- |
|  | 1♂ | 2♂ | 3♂ | 4♀ | 5♀ | 6♀ |  |  |
| 1 h | 7.50 | 7.09 | 7.51 | 7.27 | 3.61 | 3.50 | 6.08 | 1.96 |
| 2 h | 6.25 | 3.54 | 6.19 | 3.62 | 4.26 | 3.87 | 4.62 | 1.26 |
| 4 h | 5.67 | 3.51 | 5.75 | 3.40 | 3.08 | 3.01 | 4.07 | 1.28 |
| 8 h | 6.00 | 3.03 | 6.14 | 3.09 | 4.59 | 4.51 | 4.56 | 1.35 |
| 12 h | 7.21 | 3.53 | 7.35 | 3.53 | 3.54 | 3.39 | 4.76 | 1.95 |
| 24 h | 10.09 | 5.50 | 10.02 | 5.46 | 5.20 | 5.17 | 6.91 | 2.44 |
| 29 h | 7.97 | 5.13 | 7.87 | 5.28 | 6.53 | 5.92 | 6.45 | 1.24 |
| 40 h | 6.28 | 6.91 | 6.05 | 6.91 | 8.58 | 8.54 | 7.21 | 1.10 |
| 48 h | 8.57 | 6.42 | 8.30 | 6.53 | 8.87 | 8.67 | 7.46 | 1.14 |
| 72 h | 7.32 | 9.70 | 7.13 | 9.90 | 9.14 | 9.01 | 8.70 | 1.19 |
| 76 h | 8.54 | 8.03 | 8.42 | 8.08 | 10.67 | 10.48 | 9.04 | 1.21 |
| 82 h | 5.52 | 6.11 | 5.56 | 6.61 | 7.13 | 7.17 | 6.35 | 0.74 |
| 96 h | 7.10 | 5.78 | 7.28 | 6.59 | 8.00 | 7.97 | 7.12 | 0.85 |
| 106 h | 5.93 | 5.92 | 6.25 | 5.73 | 6.22 | 6.29 | 6.06 | 0.23 |
| 110 h | 6.96 | 6.39 | 7.32 | 7.39 | 7.46 | 6.49 | 7.00 | 0.47 |
| 120 h | 4.15 | 4.70 | 4.48 | 4.40 | 5.62 | 5.56 | 4.82 | 0.62 |
| 126 h | 6.30 | 7.66 | 6.50 | 7.07 | 7.35 | 7.33 | 7.03 | 0.53 |
| 132 h | 2.50 | 5.45 | 2.54 | 5.15 | 5.91 | 5.86 | 4.57 | 1.61 |
| 144 h | 2.97 | 3.09 | 3.13 | 1.69 | 6.10 | 5.78 | 3.79 | 1.75 |
| 150 h | 4.21 | 3.50 | 4.25 | 3.62 | - | - | 3.90 | 0.39 |
| 156 h | 3.05 | 3.30 | 3.16 | 3.51 | 3.42 | 3.58 | 3.34 | 0.20 |
| 168 h | 4.48 | 4.47 | 4.65 | 4.68 | 3.94 | 4.03 | 4.37 | 0.32 |
| 192 h | 4.86 | 4.84 | 5.03 | 5.16 | 4.70 | 4.74 | 4.89 | 0.18 |
| 216 h | 4.56 | 2.90 | 4.79 | 3.02 | 4.65 | 4.83 | 4.13 | 0.91 |
| 240 h | 2.94 | 2.69 | 2.99 | 2.76 | 2.90 | 2.89 | 2.86 | 0.11 |
| 264 h | 2.58 | 2.33 | 2.63 | 2.47 | 2.84 | 2.79 | 2.61 | 0.19 |
| 288 h | 1.59 | 2.24 | 1.67 | 2.85 | 1.16 | 1.59 | 1.85 | 0.60 |
| 312 h | 1.74 | 1.58 | 1.78 | 1.33 | 1.25 | 1.33 | 1.50 | 0.23 |
| 336 h | 1.46 | 1.51 | 1.48 | 1.19 | 1.22 | 1.26 | 1.35 | 0.14 |
| 384 h | 1.27 | 1.33 | 1.29 | 1.06 | - | 1.26 | 1.24 | 0.11 |
| 432 h | 1.85 | 1.35 | 1.87 | 1.08 | 1.76 | 1.81 | 1.62 | 0.33 |
| 480 h | 1.73 | 1.04 | 1.28 | 1.02 | 1.18 | 1.33 | 1.26 | 0.26 |

BQL: <LOQ

**Figure captions**

Figure S1 - The standard curve of TVM.

Figure S2 - The standard curve of IVM.

Figure S1 - The standard curve of tenvermectin.


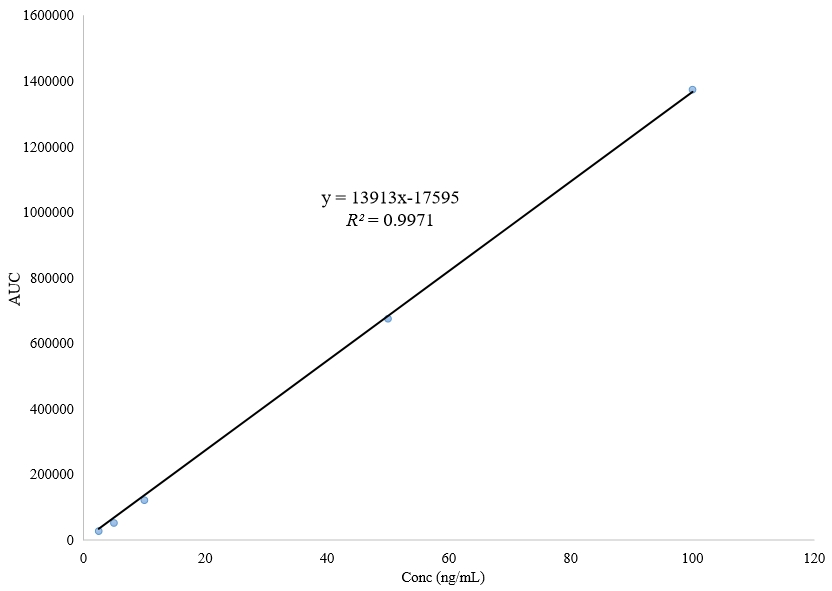


Figure S2 - The standard curve of ivermectin.


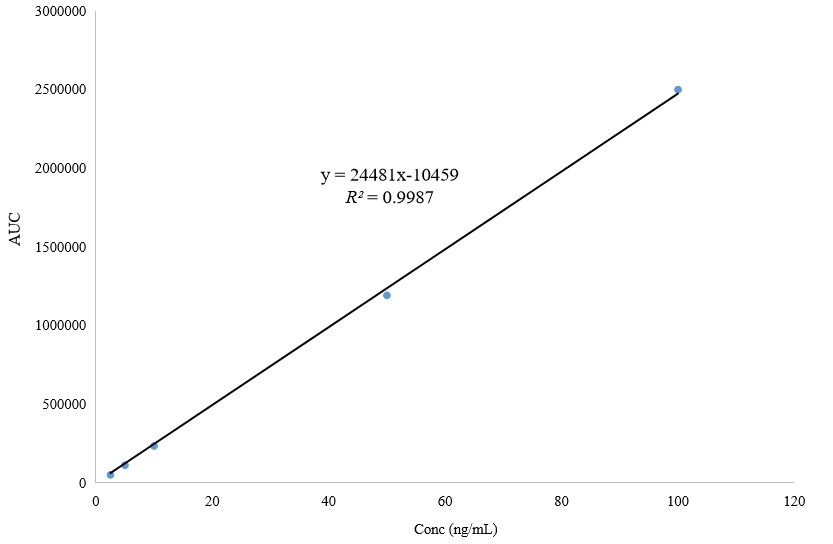

Supplement: Supplementary file 1 — Supporting Information [file VMS3-9-1211-s001.docx]
